# Combined Filling

**Published:** 1883-12

**Authors:** 


### COMBINED FILLINGS.

Robinson's Textile Tin, or any other material of like character, may be so combined with oxy-chloride or oxy-phosphate of zinc, as to make a filling that largely possesses the advantages of both. Separate the fibres and thoroughly wet them with the fluid, then mix to a paste with the powder and use like an oxychloride.

